# Psychophysiological distinctions in emotional responding: sensitivity to perceiving loss of connection

**DOI:** 10.3389/fnhum.2024.1363546

**Published:** 2024-09-09

**Authors:** Lily Seah, Bruce H. Friedman

**Affiliations:** Cognitive Neuroscience and Biopsychology, Department of Psychology, Virginia Polytechnic Institute and State University, Blacksburg, VA, United States

**Keywords:** HR, HR variance, respiratory sinus arrhythmia, sadness, Chinese, European, parasympathetic, Cyberball

## Abstract

Emotion involves oneself in relation to a subject of attention; e.g., sadness is to be sad about something/someone. This study examined emotional responses to perceiving a loss of connection from oneself. Evidence suggests that Europeans tend to perceive salient objects in the foreground, while East Asians are more likely to perceive holistically, considering the interrelationships between the context and the object. We studied how this distinction affected European Americans’ (EA) and Chinese Americans’ (CA) sensitivity to perceiving the loss of connection. Both groups were exposed to loss by playing Cyberball, a ball-tossing video game, and then watched a film clip on grief. We hypothesized that EA would respond with increasing heart rate (HR) variance around the mean when perceiving loss. CA were predicted to show no difference from controls. We also hypothesized that EA would feel sadder, in terms of decreased HR and increased respiratory sinus arrhythmia (RSA), earlier during the film clip. In total, 53 subjects were recruited, of which 40 were EA (47.5% women, age 21.08 ± 1.94 years) and 13 were CA (61.5% women, age 21.05 ± 1.74 years); 25 subjects (19 EA, 6 CA) received 2 out of 48 balls tossed in Cyberball and the controls received 10. ECG, respiration, and facial electromyography (fEMG) data were acquired. The results during Cyberball showed that EA’s HR variance relative to baseline (HR SD_c/b_) had an upward trend on perceiving loss. Contrary to prediction, CA also showed higher levels of HR variance relative to baseline. The ANOVA of HR SD_c/b_ revealed that the interaction effect of two factors, time and condition, was statistically significant (*p* = 0.009). However, as predicted, EA in the experimental condition had decreased HR and increased RSA, a sign of withdrawal in sadness, 30 to 60 s into the sad clip. fEMG data at the corrugator muscle revealed that EA activated higher peak intensity 5.5 s earlier than CA (increased 1.571 vs. 0.844). This difference, however, was not statistically significant. The evidence suggests that increased exposure to loss automatically led to increased HR variance in both groups even when subjects were informed that players were computer-generated. However, the effect was stronger on EA to increase their arousal and sensitivity to grief thereafter.

## 1 Introduction

Anyone who has enjoyed a film knows how powerful storytelling can be in moving one to identify with the protagonists and feel their emotions as the story unfolds. Obviously, emotions can be experienced in relation to real events, but one can also be moved to emote when immersed in a make-believe story. Theorists vary on whether emotions are automatic or involve self-agency (see Friedman and Thayer, 2024, for a review). At one end of the spectrum are basic emotion theories, which posit that emotions evolved from adaptations to automatically respond to prototypical antecedent events (e.g., Ekman, 1992; Izard, 1992; Panksepp, 1992; Ekman and Cordaro, 2011). At the other end are the psychological constructivist theories, which consider each instance of emotion as cognitively constructed (e.g., Russell, 2009; Quigley and Barrett, 2014; Barrett, 2017). There are also the appraisal theories, which argue that emotion is adaptive but emerges from cognitive appraisal of the environment (e.g., Lazarus, 1982, 1984; Moors et al., 2013). Further consideration of these emotion theories is beyond the scope of this study, but it is important from a temporally dynamic perspective to consider when an emotion emerges, i.e., as an immediate and automatic environmental response, as is implied in basic emotion theory, or as a subsequent process to cognition.

In this study, we viewed emotion as involving oneself *in relation to* a subject of attention, e.g., sadness is to be sad about something/someone, and anger is to be angry with something/someone. In the information processing paradigm of cognitive science, the self is framed as separate from the environment, perceiving through the internal representation of external objects as symbols. Accordingly, the primacy of cognition is often assumed. Studies typically examine emotional reactions to situations or objects as entities separate from oneself, and rarely consider how the nature of self-environment coupling affects emotion. Noted psychologist Gibson (1966, 1986) proposed a perspective that the self and the environment are a tightly coupled, inseparable pair, and Gibson argued that there are invariants, known as *affordances*, in the structure of the environment with rich stimulus information to be perceived and acted on directly. Importantly, an affordance points two ways in that it contains information about its usefulness, as well as information regarding the observer, because to perceive the environment requires “co-perception” of oneself (Gibson, 1986).

In the last decades, several researchers have developed this perspective further to better understand social, cognitive, and affective phenomena (De Haan et al., 2013; Rietveld and Kiverstein, 2014; Withagen, 2018). However, in the study of emotion, there has been limited use of the idea of “affordance,” albeit without reference to Gibson’s view on the coupling of the self-environment (Suri et al., 2018). In our study, we take a Gibsonian view of emotions as responses to changes in the self-environment coupling that require neither cognitive appraisal nor construction. This position is consistent with evidence that affect can operate independent of cognitive appraisal and can precede cognition in a chain of behavioral responses (Zajonc, 1980, 1984, 2000; cf. Lazarus, 1982, 1984). For example, emotion can be a response to a mere-repeated-exposure phenomenon, a subliminal preference developed through repeated exposure (Zajonc, 2001). Consider, then, the example of acute sadness. Autonomic nervous system (ANS) activity in sadness suggests decreased sympathetic activity, as is indicated by decreased heart rate, increased heart rate variability, decreased blood pressure, and decreased skin conductance (Kreibig, 2010, p. 401). This response pattern gives the impression that the feeling of sadness involves physiological withdrawal from the environment and decreased responsivity in the self–environment interaction. Neither the antecedent events nor the self alone is sufficient to explain the onset of emotion. An emotion arises out of a dynamic interplay between self (i.e., me, mine, and I) and affordances in a reciprocal relationship. The focus of our study is on the effects of a change in the self–environment relationship. We took a novel, dynamic approach to examine the psychophysiological and emotional effects of perceiving a loss of connection from oneself. People in one’s environment provide social affordances. A loss in the connection to others may be experienced first as a loss of connection from self. As such, this is not “social exclusion,” as this term requires the cognitive appraisal that others have acted to exclude one from the relationship. In contrast, the emotion of interest in our study is more basic than social emotions.

Note that the above propositions do not imply that appraisal or self-agency is absent. The agency can be present in receptivity. Gibson’s view dovetails with William James’s well-known definition of emotion: “… bodily changes follow directly the PERCEPTION of the exciting fact, and that our feeling of the same changes as they occur IS the emotion” (James, 1884, pp. 189–190; all caps in the original). This definition of emotion as the feeling of bodily changes captures the view that bodily arousal and associated behaviors precede, occur in parallel with, and are a necessary condition for subjective feeling. The receptive bodily response, together with the subjective feeling, could constitute a signaling system that spontaneously leads to retreat from or to advance with a specific situation or person.

We posed the question of whether there might be distinctions in perception of self and others between groups, which may imply different forms of coupling between self and the environment. For example, socially independent versus interdependent cultures may differ in this coupling. In fact, there is evidence that individuals of European descent versus those of East Asian descent differ in perception and thought about what the environment affords (Nisbett, 2003; Nisbett and Masuda, 2003; Nisbett and Miyamoto, 2005; Miyamoto et al., 2006; de Oliveira and Nisbett, 2017). Specifically, individuals of European descent tend to perceive and attend to salient objects in the environmental foreground and think in an analytic way (as in understanding by breaking down into parts) independent of context, in contrast to those of East Asian descent, who tend to perceive and attend to context and object in a holistic way. In a study comparing American and Japanese samples, subjects were shown a video clip of an underwater scene with fishes as focal objects, and other smaller animals, plants, and rocks as the background, and asked to describe what they saw (Masuda and Nisbett, 2001; Nisbett and Miyamoto, 2005). American subjects frequently first described focal (i.e., foreground) objects, whereas Japanese subjects more often began by referring to the context. Overall, Japanese subjects described 60% more information about the context than American subjects did. In another study, American subjects were more sensitive to changes in the salient objects, whereas Chinese subjects were more sensitive to changes in the context (Masuda and Nisbett, 2006).

Electrophysiological evidence is consistent with these findings. Using the N400 event-related potential (ERP), a study compared the responses of European Americans and Asian Americans, half of whom were East Asian Americans, to perceiving incongruence between emotionally expressive faces and background affective scenes (Goto et al., 2010). The N400 component is a negative response (in relation to pre-stimulus baseline) at specific scalp locations that peaks at approximately 400 ms. The amplitude of the waveform is a good measure of changes in stimuli. Asian Americans showed greater sensitivity to incongruent N400s, whereas European Americans did not show a significant difference between incongruent and congruent trials. In a further study using the error-related negativity (ERN), ERP as a neural marker for self-centric motivation, European Americans showed significant increases in ERN in a computer task on earning rewards for themselves (versus a friend), whereas East Asian/Asian Americans did not show a self-serving bias. The ERN component is a large negative response that is observed within 100 ms after an error occurs. An increase in ERN is indicative of higher motivational attention to the task at hand. The authors suggest that Asians “have a more extended self that encompasses close others as its part” (Kitayama and Park, 2014, p. 68).

A few empirical studies have compared Europeans and East Asians on the effect of culture, i.e., individualism versus collectivism or independence versus interdependence, on social exclusion. A study comparing German and Chinese found German subjects, but not Chinese subjects, showed a heart rate (HR) increase (Pfundmair et al., 2015). A further study comparing European Americans and East Asian/East Asian Americans found European Americans reported anger and sadness, whereas no significant change in emotions was found in East Asian subjects (Kimel et al., 2017). In an fMRI study, the dorsal anterior cingulate cortex (dACC), an area implicated in physical pain, was found to have increased activity in excluded subjects and a positive correlation with self-report of distress (Eisenberger et al., 2003). The authors interpreted the findings as that the brain processes social pain similarly to physical pain. A follow-up study examined whether the μ-opioid receptor gene, which morphine acts on for pain relief, can distinguish individual differences in sensitivity to rejection (Way et al., 2009). The findings revealed that A118G polymorphism, a measure of the μ-opioid receptor gene, was associated with self-report of dispositional sensitivity to rejection. In a subsample, the G allele carriers were found to have greater reactions to rejection in brain regions related to social pain and physical pain. The G allele carriers are known to require more morphine to deal with physical pain. Because proportions of social sensitivity alleles, e.g., the G allele, in cross-national populations correlated positively with the degree of societal collectivism, these researchers subsequently inferred that a high proportion of these alleles play a role in shaping the degree of social interdependencies in East Asian countries (Way and Lieberman, 2010). This suggests that distinctions in social loss between individuals of European descent and East Asian descent may have a genetic basis. However, there are psychophysiological studies that compared emotional responses between European Americans and Asian/Asian Americans that found differences in facial expression and self-report of affect, but no physiological differences (Tsai and Levenson, 1997; Tsai et al., 2000). From this theoretical and empirical background, we designed a study to examine the effects of loss of connection from oneself. Specifically, we exposed two groups of Americans, one of European descent and one of Chinese descent, to the loss in Cyberball, a ball-tossing video game. Cyberball has been used extensively to study the effects of social exclusion (Williams et al., 2000; Eisenberger et al., 2003; Williams and Jarvis, 2006; Wesselmann et al., 2012; Schmälzle et al., 2017; Williamson et al., 2018; Dewald-Kaufmann et al., 2021). A meta-analysis of 120 Cyberball studies, comprising 97% from the United States and other Western countries, and 3% from Asian countries, found the average ostracism effect to be large (Hartgerink et al., 2015).

Typically, in Cyberball studies, subjects are led to believe that they are playing with real people, even when players are actually computer-generated. In a series of Australian studies in which subjects were told the players were computer-generated, the adverse effects on self-reported sense of belonging, control, and self-esteem were as negative as other typical studies on exclusion (Zadro et al., 2004). In a Japanese study in which ERP, EEG, and facial electromyography (fEMG) were measured to examine temporal changes, negative affect as measured by fEMG at the corrugator supercilii facial muscle significantly increased on exclusion, even when subjects knew players were not real (Kawamoto et al., 2013). In the last two studies, rather than social exclusion by the computer, it is more accurate to describe the phenomenon as simply perceiving a loss of connection from oneself as no real players were involved. To better examine the effect of perceiving loss, we did not use deception in this study and both groups knew that they were playing with computer-generated players. We expected that cognitive evaluation of being socially excluded to be reduced considerably. As such, this study differs from other social exclusion studies involving real people (DeWall et al., 2011).

Considering the self-environment coupling, and evidence of distinctions in perception, we predicted that EA would respond negatively to the loss of connection in Cyberball, whereas CA would not. We reasoned that when there is sensitivity to perceiving focal objects in the foreground, there would also be increased sensitivity to perceive them in relation to oneself (as the center). A loss of connection would elicit sadness because decoupling with an affordance is felt personally. On the other hand, when sensitivity is in perceiving the field, which encompasses the context, and interrelationships with focal objects, the reference point would be diffused in the entire field, of which the self is a part. As such, loss of connection from oneself would tend not to elicit an emotional response, other than possibly certain bodily arousals or movements to counterbalance the change. To better distinguish the affective response during Cyberball, an emotion amplifier, by way of a sad film clip, was added to follow shortly after the manipulation of loss. This idea of an amplifier implies continuity in the stream of experience such that the effects of a change in the self-environment coupling during the Cyberball may propagate beyond that to affect the experience of watching the sad clip, especially when the self is perceived as the center of the experience.

In sum, our study draws on the above theoretical and empirical perspectives that fit naturally but have rarely been integrated. The aim is to inform on distinctions in emotion as a signaling system in the coupling between self and the environment. Specifically, we hypothesized that:

1. EA and CA would on average respond similarly in the control condition. However, we anticipated differences in physiological measures over time, reflecting distinctions in temporal dynamics.
2. CA in the experimental condition would show an HR decrease over time as their heart rhythms synchronized with the slower rhythm of ball-throwing from one player to another as they watched on. Apart from this measure, CA in both conditions would not show significant differences in affective and physiological responses during Cyberball and the sad clip.
3. EA in the experimental condition would have increased variability in the heart rhythm over time as measured by an increased HR variance relative to the baseline, as subjects continually anticipate a player to throw them a ball and feel a loss whenever that does not happen. Furthermore, during the sad clip, EA in the experimental condition would respond faster to reach peak intensity (positive or negative) earlier in terms of the activation of the corrugator supercilii muscle, and a simultaneous decrease in HR from enhanced cardiac vagal activation and increased respiratory sinus arrhythmia (RSA) (Kreibig et al., 2007).

## 2 Materials and methods

### 2.1 Research subjects

We obtained Institutional Review Board (IRB) approval before recruiting subjects from the Virginia Tech campus. The methods of recruitment included posting flyers on notice boards, broadcast of study in campus newsletters, and announcements in classes. Individuals who signed up were screened for these inclusion criteria: (1) EA or CA; (2) between the ages of 18 and 28 years; (3) born or raised in the USA before the age of 4 years; and (4) in good physical and mental health. Individuals with neurological conditions, e.g., stroke, or regular use of nicotine or drug abuse issues, were excluded from the study. Individuals with other significant issues in mental or physical functioning, e.g., hearing loss and color blindness, were also excluded to control for factors that may affect facial expression or subjective experience that are unrelated to the manipulation under study.

In total, 53 subjects participated in the study, of which 40 were EA (47.5% women, age 21.08 ± 1.94 years) and 13 were CA (61.5% women, age 21.05 ± 1.74 years). As the population of CA in Blacksburg is small relative to that of EA, we were not able to recruit as many CA during the period of recruitment; 86.8 and 9.4% of subjects were undergraduates and graduate students in Virginia Tech, respectively; 6 subjects had reported having medical issues. In the EA group, two had scoliosis, one had anxiety, and one had a grass-induced breathing problem, whereas in the CA group, one had a hole in the heart and another one had exercise-induced asthma.

CA subjects filled out the Suinn–Lew Asian Self-Identity Acculturation Scale questionnaire (SL-ASIA) (Suinn et al., 1992) to measure their orientation to the American culture, whereas the EA subjects filled out a modified version of the questionnaire. Both questionnaires comprise multiple-choice questions on a 5-point Likert scale ranging from 1 (very Asian) to 5 (very American) or from 1 (very European) to 5 (very American). The mean scores from questions 1 to 21 were 3.63 ± 0.23 for EA and 3.24 ± 0.33 for CA. Though the means differed, the two values did not show extreme values, such as below 3 or above 4. As all the subjects were born in the USA or entered the country as infants and were raised and educated in the USA, their language, food, entertainment, and social preferences were more alike than different.

However, responses to question 26 revealed a marked difference in self-identity as shown in Table 1; 92.5% of EA identified either basically as Americans (statement 2) or first as Americans (statement 4), while 7.7% of CA identified first as Americans. The rest of the CA (92.3%) viewed their identities as tied to being Asian, whether it is a blend of Asian and American characteristics (53.8%), Asian first (23.1%), or basically Asian (15.4%). None of the CA subjects saw themselves as basically American (statement 2), the identity most EA subjects associated with. The difference could stem from the fact that most of the EA subjects have deeper roots in the USA as indicated by responses to question 12; 75% of the EA subjects were 3rd, 4th, or 5th generation in the USA, whereas 100% of the CA subjects were 1st and 2nd generation in the country. It is noteworthy that two of the CA subjects were adopted by European American families when they were infants. One identified as an Asian American with a blend of Asian and American characteristics (statement 5), while the other identified as basically Asian (statement 1).

**Table 1:** Comparison of responses to question 26 in Suinn-Lew Survey.

Question 26—self-identity	European American (No. of responses)	Chinese American (No. of responses)
1. I consider myself basically an Asian (European) person. Even though I live and work in America. I still view myself basically as an Asian/ European person.	0	2
2. I consider myself basically as an American. Even though I have an Asian (European) background and characteristics, I still view myself basically as an American.	31	0
3. I consider myself as an Asian-American (European American), although deep down, I always know I am an Asian (European).	0	3
4. I consider myself as an Asian-American (European American), although deep down, I view myself as an American first.	6	1
5. I consider myself as an Asian-American (European American). I have both Asian (European) and American characteristics, and I view myself as a blend of both.	3	7
Total	40	13

### 2.2 Cyberball and film clips

Manipulation of loss of connection was delivered through the Cyberball video game in a between-subjects design with two conditions. In the control condition, subjects received 10 of the 48 balls thrown (20.8%). In the experimental condition, subjects received 2 of the 48 balls thrown (4.2%). These two balls were thrown to the subjects within the first 20–25 s of the game, depending on how fast the subject tossed out the first ball received. After that, no further balls were thrown to the subjects until the end of the game. Unlike the experimental condition, subjects in the control condition received balls from the other players from the start to the end of the game. However, as subjects in the control condition received fewer balls than the other players, there were periods of time when subjects had to wait longer than other players to get the next ball. In the control condition, the game lasted from 3 min 52 s to 4 min 12 s, whereas in the experimental condition, the game took 4 min 11 s to 4 min 21 s. In both conditions, the throws from one player to another were pre-determined, except for the balls thrown by the subjects.

After the Cyberball game, subjects watched a sad clip (2 min 50 s) taken from the film “The Champ.” A second film clip (2 min 54 s), which is amusing, from the film “When Harry Met Sally” was added to neutralize subjects’ negative feelings. The film clips have previously been found to reliably elicit reports of targeted emotions and associated facial expressions (Gross and Levenson, 1995).

### 2.3 Manipulation check and self-report

Subjects filled out three questionnaires after the experiment. In the first questionnaire, subjects were asked to indicate the main and other emotions experienced during Cyberball and film clips. A list of words was provided, comprising “Neutral,” “Happy,” “Angry,” “Sad,” “Contempt,” “Fear,” “Disgust,” “Surprise,” and “Others.” If subjects chose “Others,” they could describe in their own words what the emotions were. In the second questionnaire, subjects were asked to specify the proportion of balls tossed to them. This was used as a manipulation check. Putting the manipulation check after the experiment ensured that the effect of manipulation was not altered by the intervention. Finally, the third questionnaire was the SL-ASIA for CA subjects and a modified version for EA subjects.

### 2.4 Facial coding

A research assistant trained and certified on the facial action coding system (Ekman et al., 2002; Paul Ekman Group, n.d.) coded videos recorded during the Cyberball game and the sad clip. The research assistant was blind to the experimental design and did not know what the subjects were watching or doing. The emotional expressive behavioral system devised by James Gross (Gross and Levenson, 1993; Gross and John, 1997) based on FACS was used for facial coding of emotion. There was only one facial coder, so the coding results were used in a limited way as supporting data.

### 2.5 Physiological measures

The Biopac MP160 data acquisition system (Biopac Systems Inc., 42 Aero Camino, Goleta, CA 93117) with the AcqKnowledge 5.0 software was used to collect these physiological data: fEMG, ECG, and respiration. To ensure that noise and movement artifacts were minimized, subjects were asked to sit upright with a straight back and keep still. Appropriate skin preparation also helped to minimize impedance. The sampling rate was set at a high resolution of 2,000 Hz.

A Biopac EMG100C amplifier was used to measure facial muscle activity at the corrugator supercilii muscle. The corrugator supercilii is located at the medial end of the eyebrow. The muscle may be activated when one is sad or angry (Larsen et al., 2003). Data collected at this muscle location were used to infer the negative affect, intensity, and duration; 3 silver/silver chloride 4-mm reusable electrodes were used, 1 for the ground and 2 for the muscle location. The amplifier was set at a gain of 2000, and a bandpass between 10 Hz and 500 Hz. An impedance check was performed at each electrode to ensure that the impedance was below 5kΩ.

The dual wireless respiration and ECG BioNomadix module with a matching transmitter and receiver were used to detect cardiac and respiratory signals. The BioNomadix module bandlimits raw ECG data to be within 0.05 Hz to 150 Hz. This wireless module works with a modified Lead II electrode placement, one electrode on each side below the clavicle bone, and a third electrode at the lower part of the rib cage. There was an impedance check to keep the level below 5kΩ. The respiration belt transducer was placed snugly below the armpit without any slack to ensure an accurate measurement of the respiration rate.

### 2.6 Laboratory procedures

Each in-laboratory session began with a briefing on the study followed by an abstention check and the consent process. Specifically, subjects were required to abstain from consuming alcohol, caffeine, and non-prescription drugs, food, and exercise, 12 h, 6 h, and 2 h prior to the scheduled session, respectively. Then, subjects were informed in the consent form that they would play a video game against players who were computer-generated. The set-up, which took approximately 60 min, included preparing the skin for electrode placement, placing the electrodes and respiration belt, testing impedance, preparing the subject to follow computer instructions, and calibrating and testing physiological signals. When ready, the subject put on an audio headset and carried on with the experiment in the room alone. The activity of the subject was monitored through two web cameras placed on the computer monitor in front of the subject.

The experimental process was delivered through the Qualtrics platform. Each subject began the experiment with a 3-min guided relaxation exercise delivered through pre-recorded audio. Subjects chose one of two audios with the same instructions, one by a man and another by a woman. At the end of the relaxation exercise, an underwater video serving as a vanilla baseline (Jennings et al., 1992) played automatically for 5 min.

After that, the introductory page of Cyberball appeared, followed by the 4-player Cyberball game. Each icon representing a player had a different color, whereas the icon representing the subject was white. The game began with one of the other three players holding a ball. At the end of the game, subjects watched two film clips and then filled out three questionnaires. The in-laboratory session ended with a 5-min debriefing, followed by payment of $25 for participating in the study.

### 2.7 Data analyses

The physiological data extracted for analysis were time series of HR, interbeat interval (IBI), respiration rate, RSA, and root-mean-square (RMS) of corrugator muscle activity. HR and IBI were extracted directly from the raw ECG data after identifying the time of QRS peak in each cardiac cycle. For the respiration data, to make computations less intensive during filter transformations, it was resampled to 62.5 samples per second by linear interpolation. A bandpass finite impulse response (FIR) filter was then applied between 0.05 to 1 Hz. This produced a respiratory signal that was cleaner and centered on zero. The respiration rates were extracted after timings for each inhale and exhale were identified and adjusted, if necessary. RSA was extracted from the raw ECG and filtered respiration data. It was calculated by taking the difference between the maximum IBI during exhalation and the minimum IBI during inhalation. For the muscle activation data, a bandpass infinite impulse response (IIR) filter was applied between 20 and 500 Hz, followed by another bandstop IIR filter applied at 60 Hz. The output data were then converted into RMS values, and windowed at 0.3 s. The RMS value is indicative of the average “power” of the EMG signal for a given period (Merlo and Campanini, 2010; Thulkar et al., 2015).

We considered the issue of what time resolution to use to compare the time series data across groups and conditions to adequately capture the dynamic effects of the manipulation. As Cyberball is a ball-tossing game, it may appear reasonable to compare the cycles of perception and action, from perceiving a ball tossed toward the subject to the subject tossing the ball out to another player. However, as the manipulation is in the perception of *not* receiving the ball and an absence of action, this cannot be used for comparison. Instead, comparisons were made over the period shortly after subjects in the experimental condition tossed out the second ball and perceived not receiving any more balls thereafter until the end of the game. Although it may at first appear better to compare at the finest resolution, which is by heartbeat or respiration cycle, the challenge is that the finer one gets the harder it is to know whether a change arises out of a physiological or psychological nature, or an interaction of both. As subjects in the experimental condition had tossed out the second ball to another player within 30 s, we decided to apply the principle of symmetry and divided the period of the Cyberball game into multiples of 30-s segments, except for the last segment. Because subjects were exposed to a rhythm of 2 balls in the first 30 s, this measures the effects at multiples of every 30 s of not getting any ball. We maintain that aggregate averages of physiological changes from one interval to another can adequately reflect the overall effect of interactions with Cyberball. To be consistent, we also divided the sad clip into 30-s segments for comparison.

The means and standard deviations of each 30-s segment from the start of Cyberball and the start of the sad clip were extracted for HR, IBI, respiration rate, and RSA. Then, relative proportions of means and standard deviations were calculated for the period during Cyberball and the film clip after the game. The proportions were obtained by dividing the mean in a current-time value by the mean in a previous period. For example, HR_c/b_ represents the mean of heart rate during a particular time segment in Cyberball relative to the overall mean of heart rate during baseline, and HR_c/c_ represents the mean of heart rate during a particular time segment in the sad clip relative to the overall mean of heart rate during Cyberball. Each relative proportion approximates the relative change from the last period. Physiological responses were expected to be relative across time when self-environment is coupled.

For the fEMG RMS, the estimated time at which muscle contraction peaked after the start of the sad clip was determined. At the same time, an estimate of the relative increase in intensity from the first 10 s of the sad clip was also determined.

The relative proportions were compared across groups and conditions using between-subject and within-subject ANOVA. This was performed using the repeated-measures analysis function of the SPSS software. To meet the requirements for ANOVA, the normality of the proportions was assessed using Q-Q plots, and equal variances between samples were tested using Levene’s test. The level of statistical significance was set at 0.05. *Post-hoc* testing was not carried out as there were less than three categories in groups and conditions.

## 3 Results

The data of 51 subjects (38 EA and 13 CA) were analyzed after excluding 2 EA subjects. One was excluded due to software issues, and the other was excluded after revealing during debriefing that she had previously seen or played a similar video game online. All the other subjects had not seen or played Cyberball before the in-laboratory session, and none had watched the film clip that followed immediately after the video game. There were 19 EA and 7 CA in the control condition, and 19 EA and 6 CA in the experimental condition.

### 3.1 Manipulation check

Subjects answered a manipulation check question on “What was the percentage of balls thrown to you?.” The mean and standard deviation of responses to the question are shown in Table 2. The mean values in each condition are close to the actual, which is 20.83% for the control condition and 4.16% for the experimental condition. The mean values between the two conditions in all cases were statistically significant with the *t*-statistics approaching zero (t(48) = 10.05, *p* = 1.07×10^−13^, European Americans, t (35) = 7.97, *p* = 1.11×10^−9^, Chinese American t(11) = 7.21, *p* = 8.65×10^−6^).

**Table 2:** Statistics of responses to question on “% of balls thrown to you”.

Mean	Overall		European Americans		Chinese Americans	
	Control	Experimental	Control	Experimental	Control	Experimental
Mean	21.95	6.20	22.44	6.62	20.63	4.92
SD	5.95	5.06	6.47	5.54	4.37	3.28

Retrospective self-reports of subjective experience during Cyberball show that, in the control condition, 88% of subjects reported neutral or positive emotions, whereas in the experimental condition, 88% of subjects reported negative emotions. This is consistent with subjects’ estimates of the percentages of balls received.

### 3.2 Cyberball

Though the affective response was not the main consideration during Cyberball, facial coding of emotion revealed two notable observations. First, in the control condition, the most prominent negative emotion coded in EA subjects by proportion and total duration was sadness; 26% of EA displayed sadness on the face lasting between 19 and 156 s in total. None of the CA subjects in the control condition displayed sadness. In addition, 71% of CA subjects and 21% of EA subjects displayed short aggregate durations of anger and/or contempt lasting no more than 12 s in total; 1 CA subject who displayed anger and/ or contempt for a total duration of 135 s is an outlier. The negative emotions coded in both groups show that both were sensitive to even a slight imbalance in the number of balls thrown at them. In this case, it was 10 out of 48 balls thrown in total, 2 balls short of a perfect balance between 4 players. Second, 42% of EA subjects in the experimental condition displayed anger and/or contempt lasting 18 to 163 s in total; 100% of CA subjects in the experimental condition displayed anger and/ or contempt of much shorter duration with only one exceeding 18 s in total, at 24 s. The emotion coded on the face of CA was the same one and of similar short duration for both conditions.

**Figure 1:**
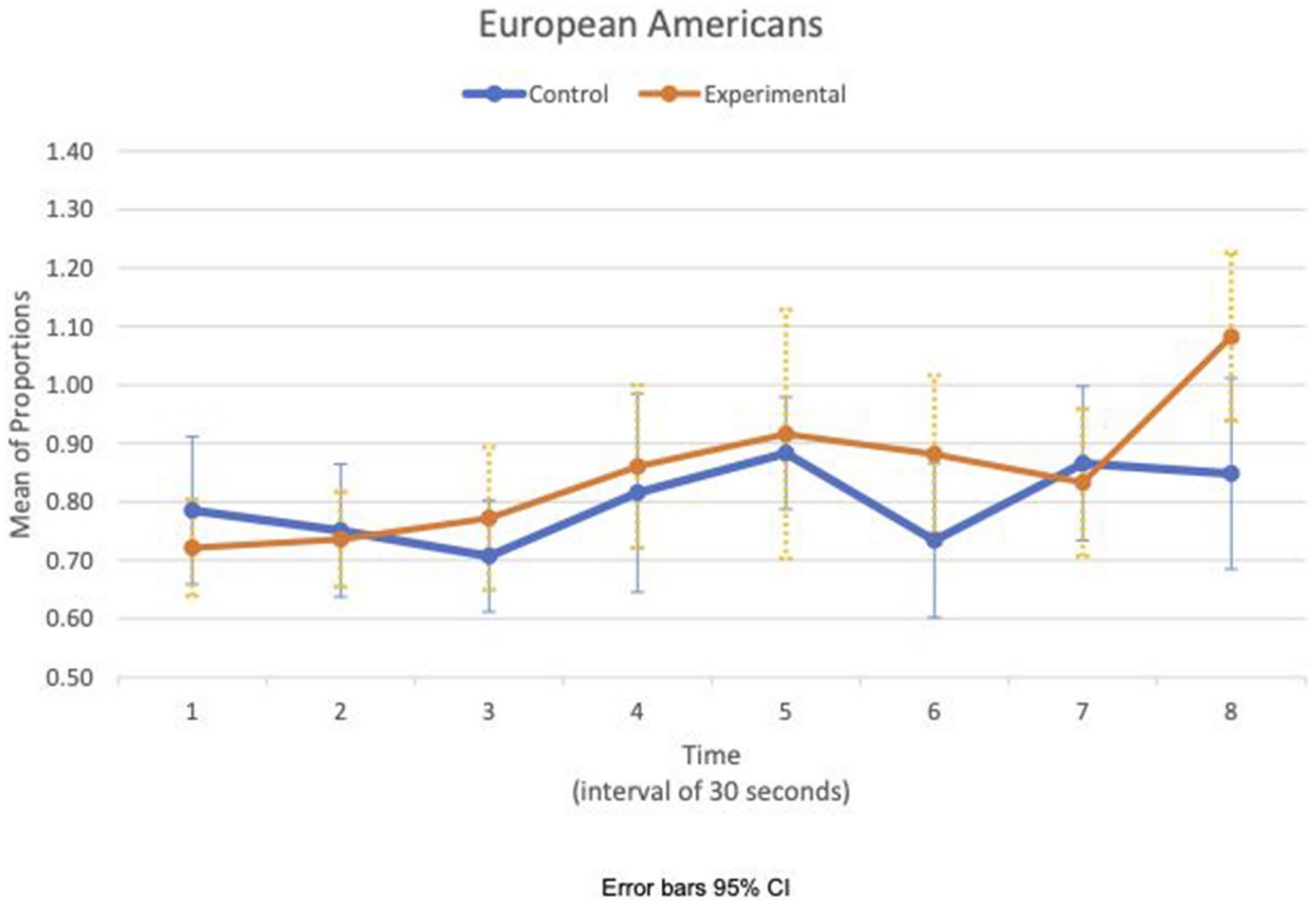
European American—means plot for HR SD_c/b_ over time (Cyberball).

**Figure 2:**
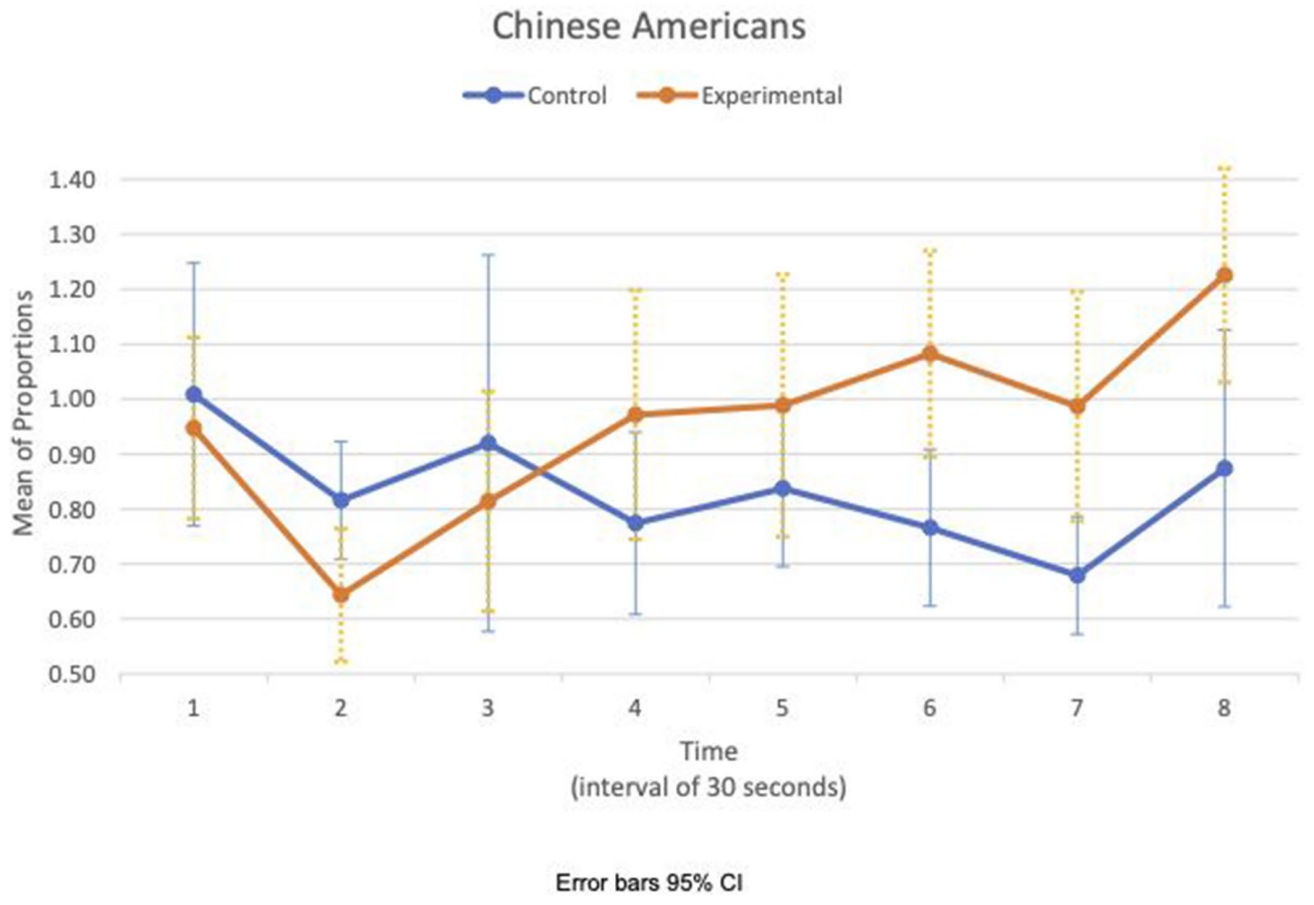
Chinese American—means plot for HR SD_c/b_ over time (Cyberball).

Between- and within-subject ANOVAs of physiological measures revealed that time had a significant effect in all measures, except RSA mean_c/b_, which had the condition as a main effect (*F*(1,47) = 4.85, *p* = 0.033, η^2^ = 0.09). Importantly, interaction effects of two factors, time and condition, are statistically significant in HR SD_c/b_ (*F*(6.6, 308.8) = 2.80, *p* = 0.009, η^2^ = 0.06) and IBI SD_c/b_ (*F*(6.7, 313.5) = 2.07, *p* = 0.049, η^2^ = 0.04). Furthermore, the interaction effects of three factors, time, condition, and group, are statistically significant in the respiration mean_c/b_ (*F*(5.8, 270.7) = 2.63, *p* = 0.019, η^2^ = 0.05). Figures 1, 2 show variations of HR SD_c/b_ for the EA and CA subjects in both conditions, respectively. Figure 1 shows that the variability of HR around the mean relative to the baseline of EA subjects in the experimental condition (HR SD_c/b_) has an upward trend. Contrary to our prediction, Figure 2 shows that HR SD_c/b_ of CA subjects in the experimental condition also has an upward trend, which increased to higher levels than EA subjects in the same condition.

**Figure 3:**
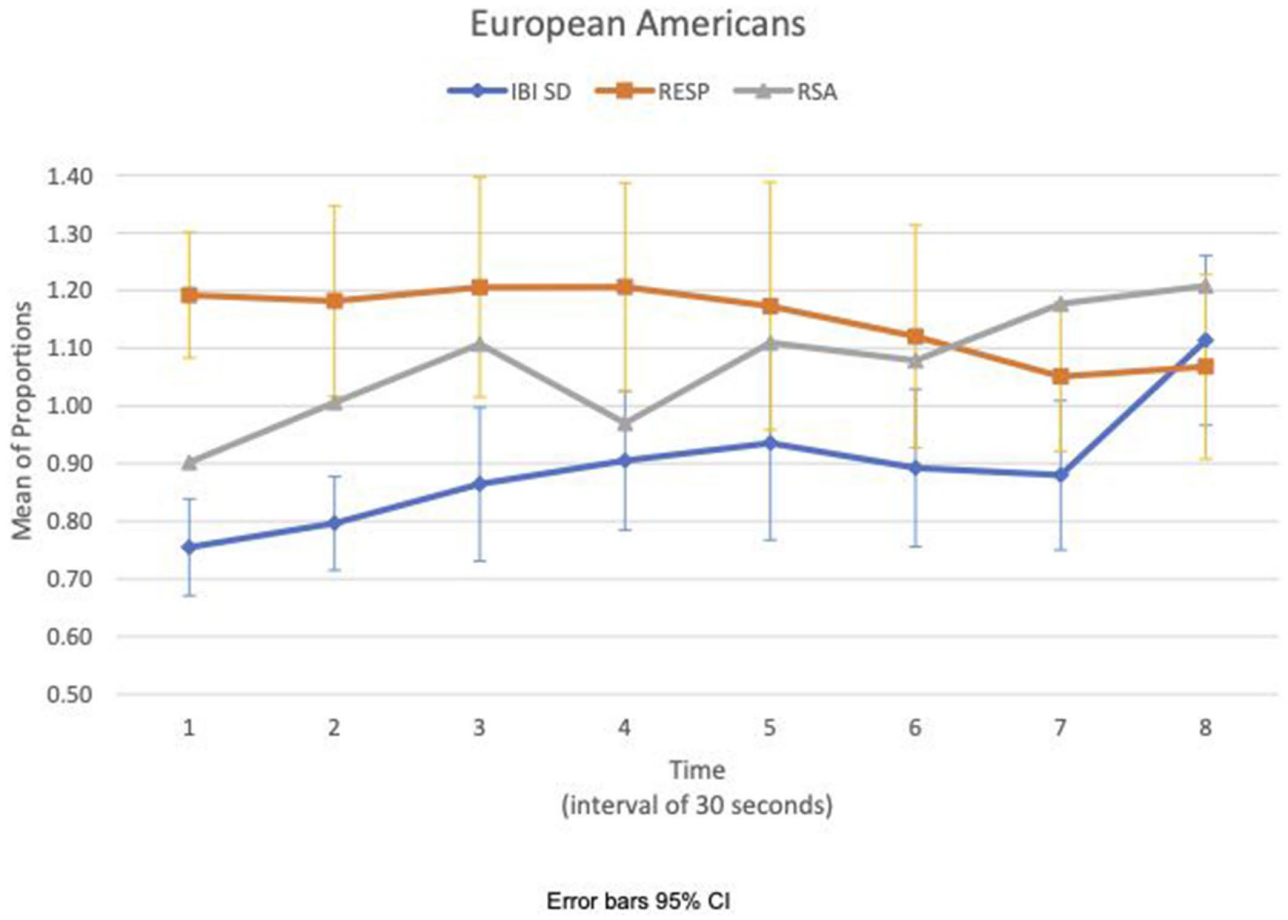
European American—covariation of RSA mean_c/b_, IBI SD_c/b_ and respiration mean_c/b_ over time (Cyberball).

**Figure 4:**
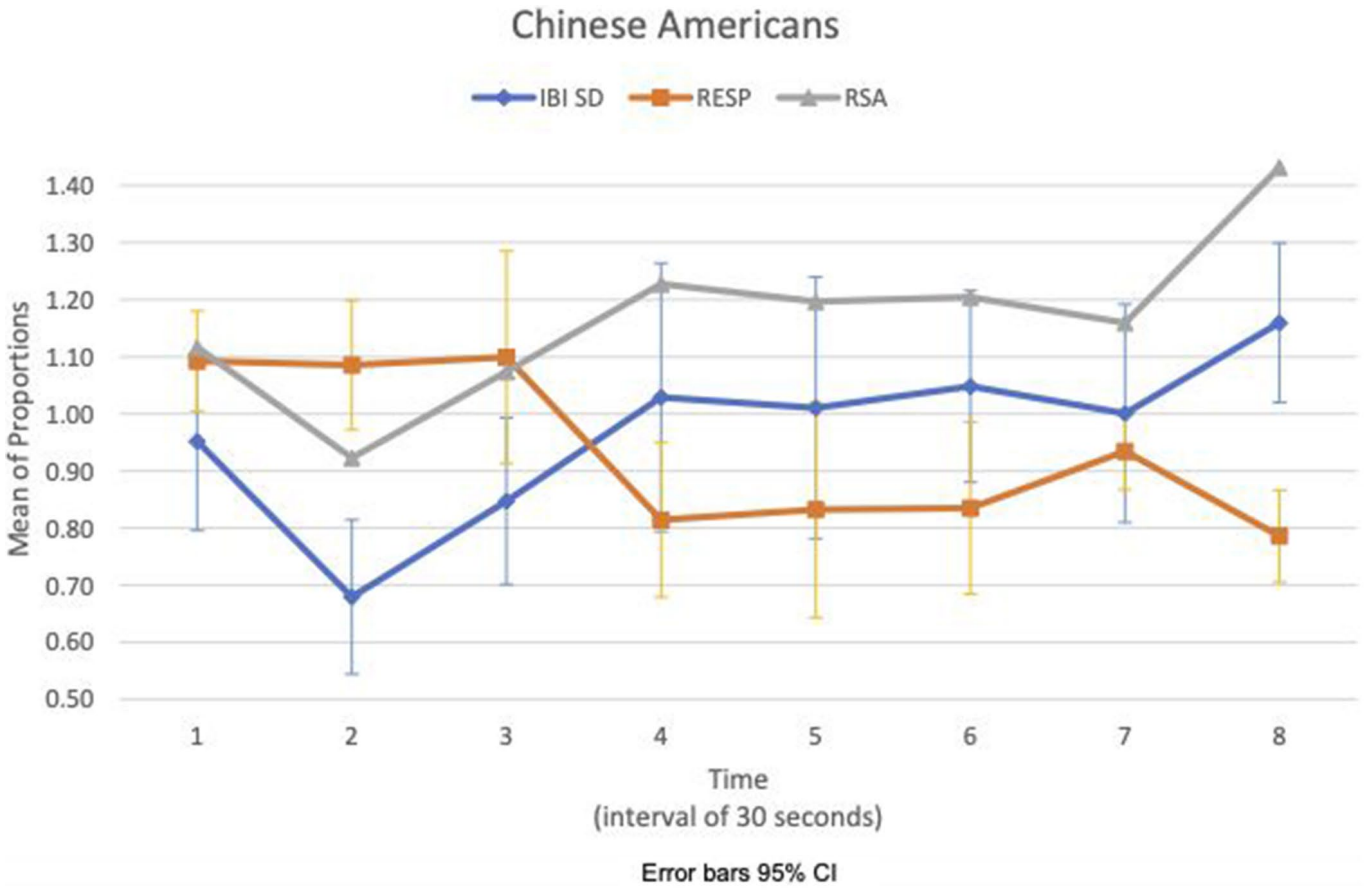
Chinese American—covariation of RSA mean_c/b_, IBI SD_c/b_ and respiration mean_c/b_ over time (Cyberball).

We examined the covariation of RSA mean_c/b_, IBI SD_c/b_, and respiration mean_c/b_ for EA and CA subjects in the experimental consition as shown in Figures 3, 4, respectively. There was no discernible pattern of covariation between RSA mean_c/b_, IBI SD_c/b_, and respiration mean_c/b_ of EA subjects as shown in Figure 3. Figure 4 shows that the RSA mean_c/b_ and IBI SD_c/b_ of CA subjects changed dynamically in the same direction throughout the video game. Respiration mean_c/b_ remained relatively constant in the first minute of the game and then changed in unison in the opposite direction with RSA mean_c/b_ and IBI SD_c/b_ thereafter.

### 3.3 Sad clip

Most subjects (88% control, 76% experimental) reported sadness as the main emotion when watching the sad clip. There were no distinguishable differences in the main emotions reported between both groups in each condition.

From the fEMG RMS time series, the time to peak intensity and increase in muscle contraction relative to baseline were determined. Cross-referencing with self-report, facial coding, and debriefing data were done to check that the corrugator muscle was activated in sadness. The data of two subjects, one EA in the experimental condition and one CA in the control condition, were not extracted because one reported feeling disgust (as the main emotion) at the sight of blood shown in the first 30 s of the clip and the other reported sadness as the main emotion, but there was no variation in the fEMG time series, possibly due to a technical issue in the fEMG recording.

After adjustments by removing three outliers (two EA and one CA in the control condition), the time to peak intensity and increase in muscle contraction relative to baseline is shown in Table 3. EA in the experimental condition reached average peak muscle contraction 109.1 s into the sad clip, which was 4.8, 5.5, and 9 s earlier than EA in the control condition, CA in the experimental condition, and CA in the control condition, respectively. EA in the experimental condition also had, on average, greater muscle contraction at 157.1% than 84.4%, 76.3%, and 69.5% for the CA in the experimental condition, CA in the control condition, and EA in the control condition, respectively. Multivariate ANOVAs on the adjusted data show that the differences in means were not statistically significant. However, separate one-sided *t*-tests on the means of an increase in muscle contraction for each group show that the average muscle contractions in the experimental condition are significantly greater than that of the control condition in the EA group (t(33) = 2.07, *p* = 0.023), but not for the CA group (t(9) = 0.14, *p* = 0.446).

**Table 3:** fEMG—comparison of means after adjustment.

Mean	European Americans		Chinese Americans	
	Control	Experimental	Control	Experimental
Time to peaked intensity from baseline (seconds)	113.9	109.1	118.1	114.6
Increase in intensity relative to baseline	0.695	1.571	0.763	0.844

Between-subject ANOVAs of physiological measures revealed that group was the main effect for HR mean_c/c_ (*F*(1,47) = 11.51, *p* = 0.001, η^2^ = 0.20) and condition was the main effect for RSA mean_c/c_ (F(1,47) = 15.53, *p* < 0.001, η^2^ = 0.25). There was no interaction effect between the group and the condition. Within-subject ANOVAs showed time as the main effect for HR mean_c/c_ (*F*(3.7, 175.1) = 4.16, *p* = 0.004, η^2^ = 0.08).

**Figure 5:**
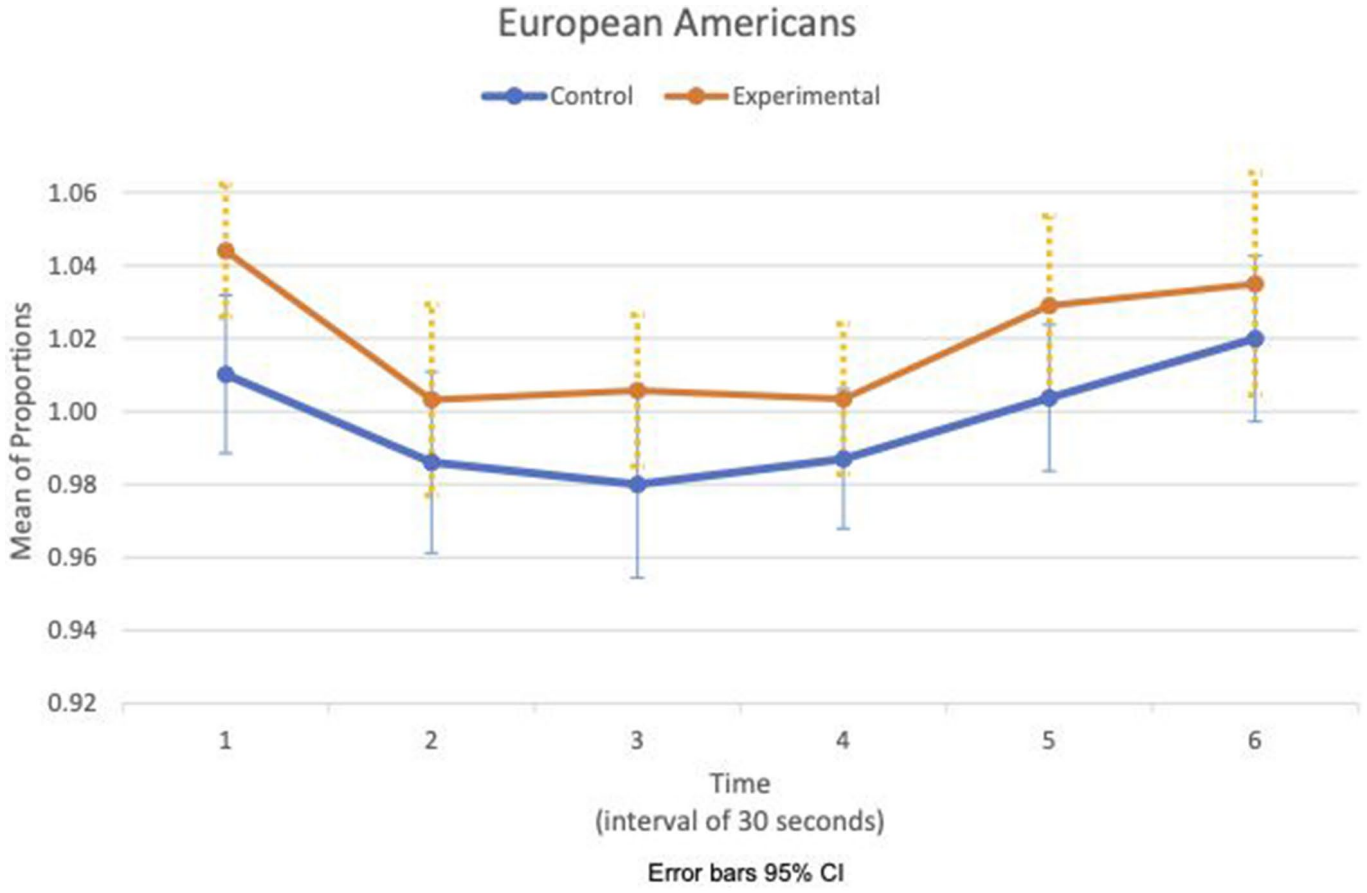
European American—means plot for HR mean_c/c_ over time (clip).

**Figure 6:**
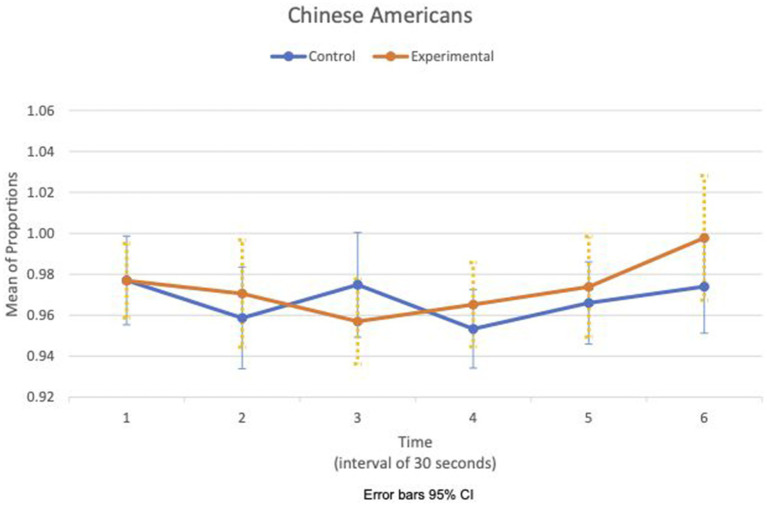
Chinese American—means plot for HR mean_c/c_ over time (clip).

**Figure 7:**
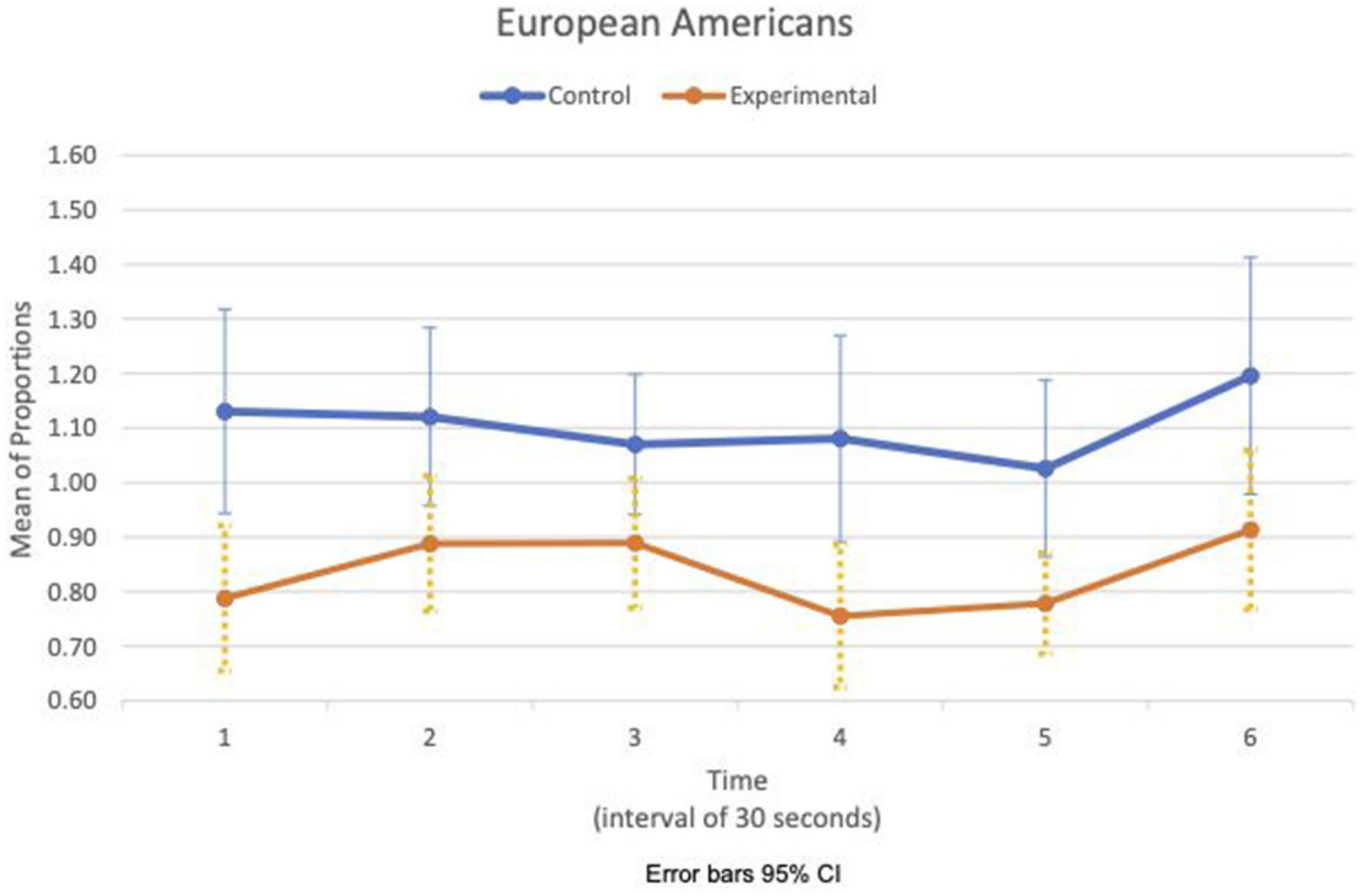
European American—means plot for RSA mean_c/c_ over time (clip).

**Figure 8:**
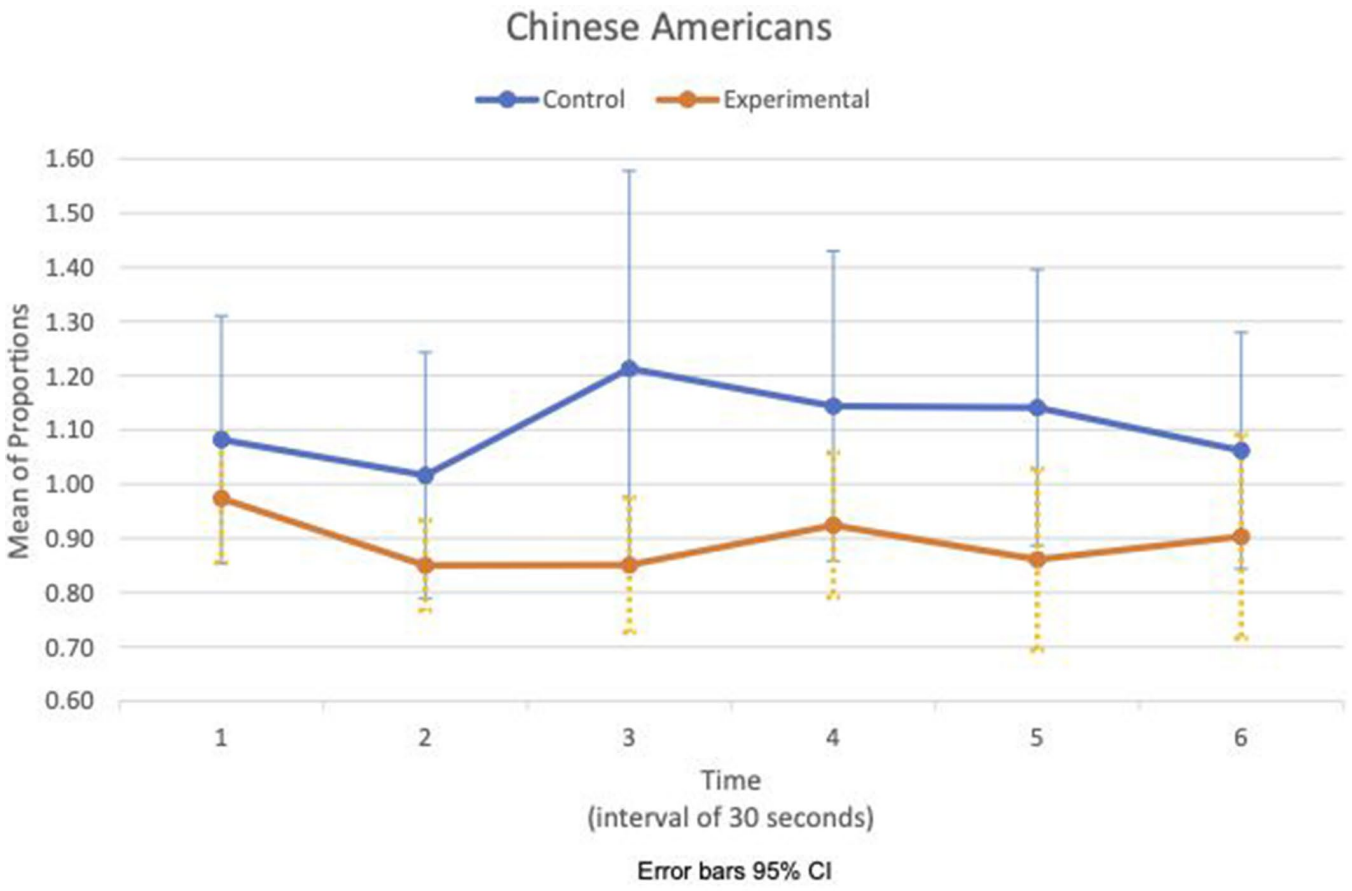
Chinese American—means plot for RSA mean_c/c_ over time (clip).

**Figure 9:**
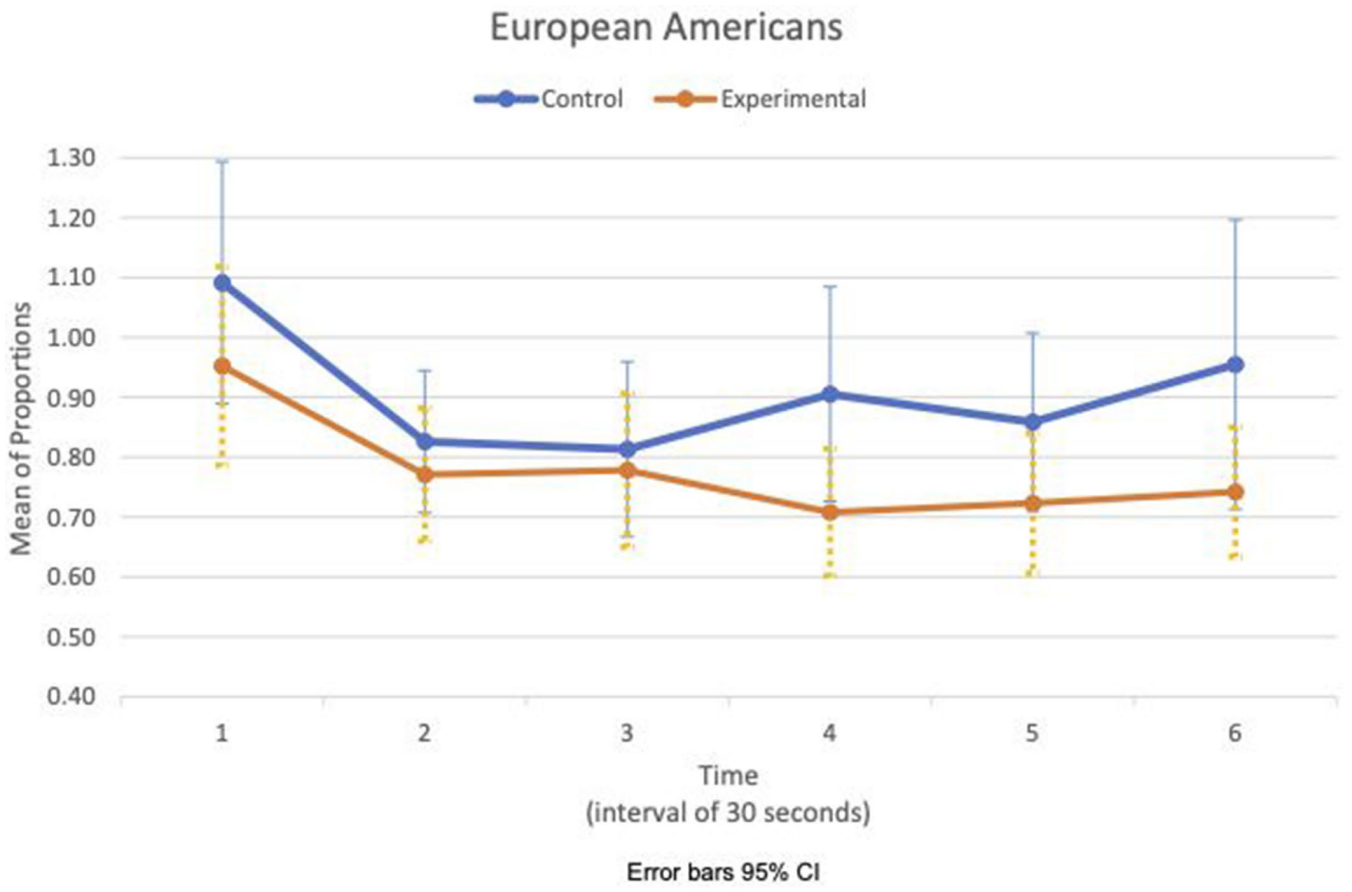
European American—means plot for HR SD_c/c_ over time (clip).

**Figure 10:**
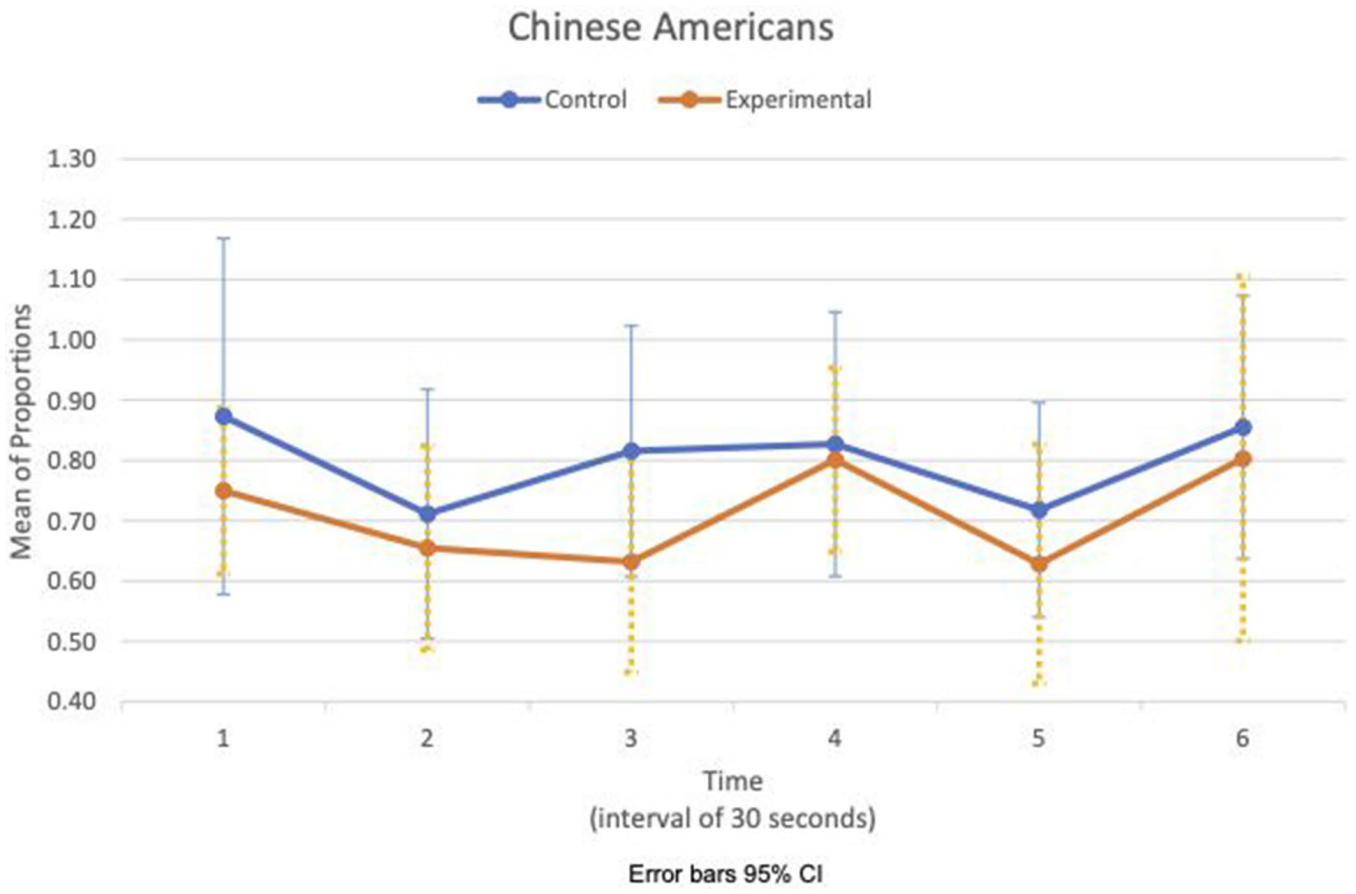
Chinese American—means plot for HR SD_c/c_ over time (clip).

Figures 5, 6 show the means plot for HR mean_c/c_ for EA and CA, respectively. Figures 7, 8 show the means plot for RSA mean_c/c_ for EA and CA, respectively. The figures revealed that EA in the experimental condition is the only group with a simultaneous decrease in HR mean_c/c_ and increase in RSA mean_c/c_ in the 2nd time interval, which is between 30 and 60 s into the sad clip. Furthermore, a comparison of Figures 9, 10 shows that EA in the experimental condition was also the only group that sustained a decrease in HR SD_c/c_ until the 4th time interval when muscle contraction peaked. After that, both the mean HR and the HR variance increased until the end of the clip.

## 4 Discussion

To summarize, the results in terms of the hypotheses: Hypothesis 1 was supported; between-subject ANOVAs of physiological measures did not reveal significant group differences in the control condition. Hypothesis 2 was not supported; within-subject ANOVAs of HR mean_c/b_ in the CA subjects in the two conditions had time as the only main effect. CA subjects in the experimental condition responded to the loss of connection not by way of a decrease in HR mean_c/b_, but by a significant increase in HR SD_c/b_. Other physiological measures in the CA subjects that were significantly different between the two conditions were IBI SD_c/b_, respiration mean_c/b_, and RSA mean_c/b_. Hypothesis 3 was partially supported; within-subject ANOVA revealed that EA subjects in both conditions showed significant differences in HR SD_c/b_ during Cyberball. As predicted, the HR variance of EA subjects in the experimental condition increased over the course of the video game. In addition, IBI SD_c/b_, respiration mean_c/b_, and RSA mean_c/b_ also differed significantly between the two conditions. The results during the sad clip provide further albeit tentative evidence in support of hypothesis 3.

It is of note that there was no deception in the study. A pre-programmed movement of a ball from one player to another was enough to elicit bodily and subjective responses from subjects to not seeing the ball come to them after making the rounds through the other players. Hence, although the game was not real (i.e., the other players were not actual human subjects), subjects experienced a loss of connection from themselves as evidenced by self-reports of subjective experiences during Cyberball. Evidence in the study suggests that responses were automatic. However, HR variance increased over time with exposure to loss for a few minutes, instead of habituating to the manipulation. This suggests that responses may not be hardwired. Rather, they may operate through a more flexible mechanism linking the self and the environment. Our findings are consistent with a series of Cyberball studies in which subjects also knew they were playing against a computer (Zadro et al., 2004). The authors concluded that the aversive responses were automatic. Another similar Cyberball study found that fEMG at the corrugator facial muscle increased significantly in the excluded condition (Kawamoto et al., 2013). The authors argued that this effect is evidence of a neurobiological foundation for exclusion. Although these findings are consistent with ours, we have framed our study differently: We posit that responses arose from a loss of connection from oneself, rather than social exclusion. The increase in HR variance over time with exposure to loss supports this position. If it was social exclusion from a computer, subjects would be expected to habituate to the manipulation. Because the impact of the manipulation was at a very basic level (i.e., a loss from self), HR variance continued to increase until the end of the game. This result suggests that responses may operate through a flexible mechanism linking self and the environment, rather than being rigidly hardwired.

The heart functions as a bio-oscillator, beating rhythmically as a part of a dynamic system that encompasses self and environment (West, 1990). The perception of another rhythm, i.e., a ball tossing among the other three players without reaching the subject, may have enhanced the variance of the oscillating heart. Neural entrainment between external stimuli and physiological responses has been noted across many domains (Rosenblum and Pikovsky, 2003). The heart may have been responding to the decoupling of self with the environment. Although both groups exhibited the same response, there were distinctions in their physiological and affective responses that suggest distinctions in the self-environment coupling.

For the CA, the effect of loss might be contained physiologically during Cyberball, thus not amplifying any affective response to the sad clip. During Cyberball, CA’s covariation of IBI SD_c/b_ and RSA mean_c/b_ in the same direction, and IBI SD_c/b_ and respiration mean_c/b_ in the opposite direction from the third time segment may suggest mutual regulation of vagal, respiration, and cardiac responses to stabilize the increased HR variance. Analogous effects have been observed in the co-activation of both cardiac sympathetic and vagal inputs to stabilize heart rate changes (Quigley and Berntson, 1990). A similar pattern of covariation across measures did not show up in EA in the experimental condition during Cyberball.

During the sad clip, the affective and physiological response of EA in the experimental condition differed from the other group and condition. Between the 1st and 2nd time segments, all groups and conditions had decreased HR mean_c/c_ and decreased HR SD_c/c_, suggesting that cardiac activity had slowed down. RSA increased only in EA in the experimental condition. An increased RSA implies that the difference in IBIs widened between inhalation and exhalation, possibly from a larger increase in IBI during exhalation than during inhalation. Given that both heart rate and its variance decreased, this increase in RSA may come from an early physiological response of withdrawal involving dual activation of two rhythmic systems, respiration and cardiac, simultaneously. EA in the experimental condition was also the only group in which the withdrawal was sustained until the third time segment when RSA decreased in concert with a decrease in HR variance. By the next time segment (fourth), the expression of sadness intensified in the face, and the corrugator muscle contraction peaked. The evidence suggests that the effect of loss may be stronger on the EA to increase their arousal and sensitivity to a sad clip thereafter.

There were limitations in the study that should be acknowledged. First, the small sample size for CA limited the statistical power of the analyses. Second, the European child actor playing the protagonist in the sad clip may have a differential effect on the responses of EA and CA in the experimental conditions. EA may be more responsive to an actor of the same ethnic group. However, as between-subject ANOVA showed that condition was a main effect in RSA (and not group), this aspect may not have had a significant impact on the results. Third, the Cyberball screen design, with four icons and a ball against a black background, may be too sparse and not sufficiently real to reveal full distinctions in perception in naturalistic settings. Fourth, the subjects were college students in their twenties. The results reflect the characteristics of this sample population and may not be generalizable to European Americans and Chinese Americans in general. These limitations can be addressed in future research by expanding the subject pool and enhancing the ecological validity of the stimuli.

In sum, our study indicates that ethnic differences in the Gibsonian construct of affordances may affect how different ethnic groups perceive and respond to loss of connection. Such differences may be revealed experimentally via a dynamic approach to the data analysis, which takes the temporal course of physiological response into account. Our study can serve as an initial demonstration of this empirical approach, which can help in the understanding of ethnic differences in affective processing.

## Data availability statement

The raw data supporting the conclusions of this article will be made available by the authors, without undue reservation.

## Ethics statement

The studies involving humans were approved by Virginia Tech Institutional Review Board. The studies were conducted in accordance with the local legislation and institutional requirements. The participants provided their written informed consent to participate in this study.

## Author contributions

LS: Conceptualization, Data curation, Formal analysis, Funding acquisition, Investigation, Methodology, Project administration, Resources, Supervision, Validation, Visualization, Writing – original draft, Writing – review & editing. BF: Funding acquisition, Resources, Writing – review & editing, Conceptualization, Supervision, Methodology.
